# Modifications to the Roter Interaction Analysis System (RIAS) coding scheme: A scoping review

**DOI:** 10.1016/j.pec.2026.109566

**Published:** 2026-02-27

**Authors:** Alejandra Martinez-Pereira, Sachi Badola, Lisa A. Mistler, Renata Yen, Nsomma Alilonu, W.Moraa Onsando, Salar Khaleghzadegan, Paul J. Barr

**Affiliations:** a Center for Technology and Behavioral Health, Lebanon, NH, USA; b The Dartmouth Institute for Health Policy & Clinical Practice, Lebanon, NH, USA; c Dartmouth Health, Lebanon, NH, USA; d Dartmouth College, Hanover, NH, USA

**Keywords:** Roter Interaction Analysis System (RIAS), Communication analysis, Coding modifications, Health communication

## Abstract

**Objective:**

The Roter Interaction Analysis System (RIAS) is a widely used coding scheme for analyzing communication in clinical encounters. Researchers often modify RIAS to fit the specific aims of their studies, but the process and rationale for modification are unclear. Without a standardized approach to modifications, it is challenging to interpret RIAS findings and benchmark results across studies. This scoping review aimed to identify and describe published RIAS modifications.

**Method:**

We searched PubMed, CINAHL, Scopus, and Web of Science for empirical studies reporting adaptations of RIAS. A narrative synthesis summarized modifications, including additions, reductions, or combinations of codes.

**Results:**

Of 551 articles identified, 27 met the inclusion criteria. While based on the original 40 mutually exclusive RIAS codes, most studies reported modifications to the coding framework. In total, 159 new codes across modified codebooks. Most newly added codes were task-focused (71%), particularly question-related. Additionally, five studies reduced and combined codes, five combined codes only, and one reduced codes. The main reasons for modification were: scope expansion, study setting, and practical considerations. Most added codes were introduced to adapt the RIAS to specific study settings. In contrast, reduced codes were often implemented for practical considerations. The majority of papers did not document their approach to modification or assessment of the modified tool.

**Conclusions:**

Shortening RIAS was a common modification, suggesting a need to revisit and streamline the coding system to enhance usability. Inconsistent documentation of modifications challenges the reproducibility and interpretation of results.

**Practice implications:**

Following established research methods and conducting psychometric testing on modified tools is critical to ensure reliability and validity. These practices improve research quality, comparability, and transparency, making findings more robust and applicable across studies.

## 1. Introduction

High-quality patient-centered communication during clinic visits is associated with improved patient satisfaction and better health outcomes [1]. To detect the occurrence of high-quality patient-centered communication, researchers have developed a range of tools to measure the quality of the visit interaction using both patient-reported surveys and trained observers who review the visit interaction using a structured coding manual [2]. A benefit of using coding manuals is that trained raters can carefully review the entire visit, and specific feedback can be provided to clinicians and patients regarding their communication, things that have gone well, and areas for improvement. With advances in AI, automated schemes for conducting this analysis are possible, yet these innovative approaches are only as good as the systems on which they are based [3].

The Roter Interaction Analysis System (RIAS) is a well-recognized coding scheme for analyzing communication in clinical encounters [4]. Constructed from social exchange theories, RIAS was developed in 1977 to assess patient-healthcare provider dynamics in socio-emotional and task-focused communication. RIAS consists of 40 mutually exclusive and exhaustive categories acknowledging the importance of dialogue in shaping the therapeutic relationship between the patient and provider. The codes used in RIAS can be organized into two broad categories of socioemotional (i.e., expressions of concern) and task-focused exchange (i.e., asking questions about physical health) [4]. Each discrete statement that captures a complete thought is labeled with a single code by an independent trained rater.

The development of RIAS was a seminal moment in healthcare communication. Since its development, RIAS has been used in over 75 communication studies across a diverse range of settings, including primary care, specialist care, genetic counseling, veterinary practice, and a growing number of additional contexts [4,5]. RIAS provides a detailed categorization of patient and provider medical dialogue and is reported to have good reliability and predictive validity across various medical contexts [4]. The system is also efficient and practical in terms of training coders on how to apply codes and system mastery.

A systematic review [5] of communication studies involving health professionals and patients, focusing on observational or experimental applications of the RIAS methodology, found that RIAS has been effective in establishing causal links between verbal components of professional communication and patient health outcomes. One of the most common applications of RIAS has been to examine the association between communication and patient satisfaction.

Despite its broad use, RIAS has also faced criticism. Common critiques include not capturing all potential domains of patient-centered communication, particularly those related to the development of tailored, patient-centered care plans [6,7]. RIAS involves independently coding participant utterances, missing the sequence of actions and details of non-verbal behaviors, which are critical to communication [8]. Further, RIAS studies are built on an existing template of general features in medical interactions; it is challenging to apply RIAS in different contexts, such as patient-pharmacist visits, without clearly adapting RIAS and adding new subcategories to exclusive topics and agendas discussed in the new context [8].

To address these challenges, many authors report adapting RIAS to address critiques and fit with their particular tool application. However, if modifications are done inconsistently, it can be difficult to standardize results and produce robust, reliable evidence. Understanding and documenting these adaptations is critical for ensuring that findings from RIAS studies can be interpreted correctly and applied in broader research and policy contexts. The objective of this scoping review was to identify and describe published modifications of RIAS. The result of this review can help better understand how RIAS has been applied, modified, and provide a critical review of the process by which researchers modify well-established tools.

## 2. Methods

### 2.1. Review protocol

We performed a scoping review guided by Arksey & O’Malley [9], including: identifying the research question, identifying relevant studies, study selection, charting the data, summarizing, and reporting results. We followed the PRISMA extension for scoping reviews (PRIS-MA-ScR) [10].

### 2.2. Study eligibility criteria

We included studies published in English that specifically mentioned making a modification to RIAS. This included adding new codes, reducing codes, or combining codes into new categories, as well as modifications related to translating original RIAS codes into another language.

### 2.3. Search terms and databases

Medical Subject Headings (MeSH) terms and keywords were combined using Boolean operators to refine the search strategy across four domains: “Roter Interaction Analysis System” AND (“adaptation” OR “modification” OR “variation”). We conducted searches in PubMed, CINAHL, Scopus, and Web of Science for articles published from database inception to August 2025. Additionally, we reviewed the reference lists of included studies to identify literature not captured in our database search.

### 2.4. Study selection

The records were imported into Rayyan (Rayyan Systems Inc., Cambridge, Massachusetts, USA), and two reviewers (SB and NA) screened all titles and abstracts independently, only excluding those that met exclusion criteria. If a determination of eligibility could not be made during the title or abstract screening, the paper proceeded to full paper review. The same reviewers screened the full text for eligibility. Disagreements between coders were resolved with a third coder (AM-P).

### 2.5. Data collection

Two independent reviewers (SB and NA) extracted data into an Excel spreadsheet, including the study characteristics (year, country, setting, methodology), research question and aims, reasons for adapting and modifying RIAS, study design, codes used in the final adapted version, and evaluations of the adapted coding scheme (ie, validity, reliability, etc). Data was extracted on the reason (if provided) for adapting the original RIAS scale and the final adapted version of the RIAS process of modification.

### 2.6. Synthesis and analysis

We conducted a narrative synthesis of the extracted data to summarize and report modifications, including: 1) a description of the modification process, 2) rationale for the modification, 3) description of the modification made (added, deleted, combined codes), 4) a categorization of newly added codes into thematic areas, and 5) any psychometric evaluation of the modified scheme.

## 3. Results

### 3.1. Description of studies

#### 3.1.1 Results of search

**Fig. 1.**
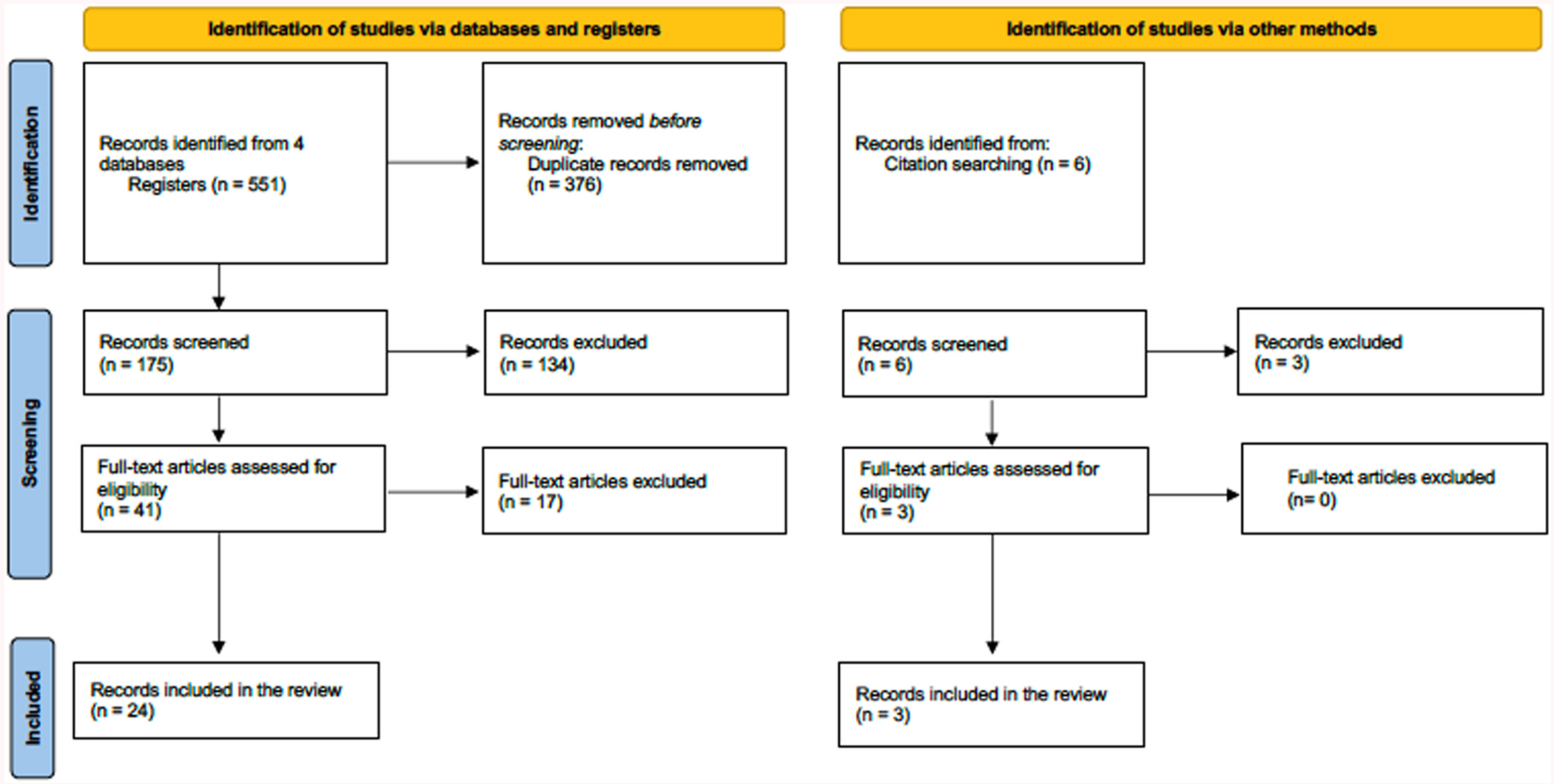
Prisma flowchart.

In total, 551 articles were found across the four databases, and 376 were excluded due to duplication. Of the remaining 175 papers, 134 did not meet the inclusion criteria and were excluded. The full text was screened for the remaining 41 papers, which resulted in 24 papers that met our inclusion criteria. We identified three additional papers through reviewing the reference lists of these papers for a total of 27 papers to include in this review (Fig. 1).

All studies were reported in English. Twelve (44.4%) were conducted in the United States, seven (25.9%) in the Netherlands, two each (7.4%) in Canada and England, and one study each in Germany, Norway, Tanzania, Italy, and Indonesia. The majority (n = 20, 74.0%) were conducted in specialty care settings - such as oncology, chronic pain, and antenatal care - while seven (25.9%) took place in general medical settings. All 27 included studies employed observational designs (Table 1).

### 3.2. Modification to RIAS

Overall, the modification processes for RIAS were inconsistently described and often lacked detail. Only one study offered a comprehensive explanation of the modification procedure [24]. Two articles described a close collaboration with RIAS experts [35,36] or were modeled on a previously published modification without providing more details [14]. The rationale for the RIAS modification, where provided, modifications made, and process used are described in Table 2.

#### 3.2.1. Rationale and purpose to modify RIAS

The main reasons cited for modifying RIAS fell into three broad categories (Table 2): for the study setting (e.g., for telemedicine encounters [19], nursing practitioner [13]), scope expansion to capture additional communication elements, and practical considerations (e.g., maximizing the system’s ability to capture patient questions [22]). Notably, ten studies (37.0%) did not justify modifying RIAS [10,12, 14–17,20,25,28,29,34].

#### 3.2.2. Type of modification

**Fig. 2.**
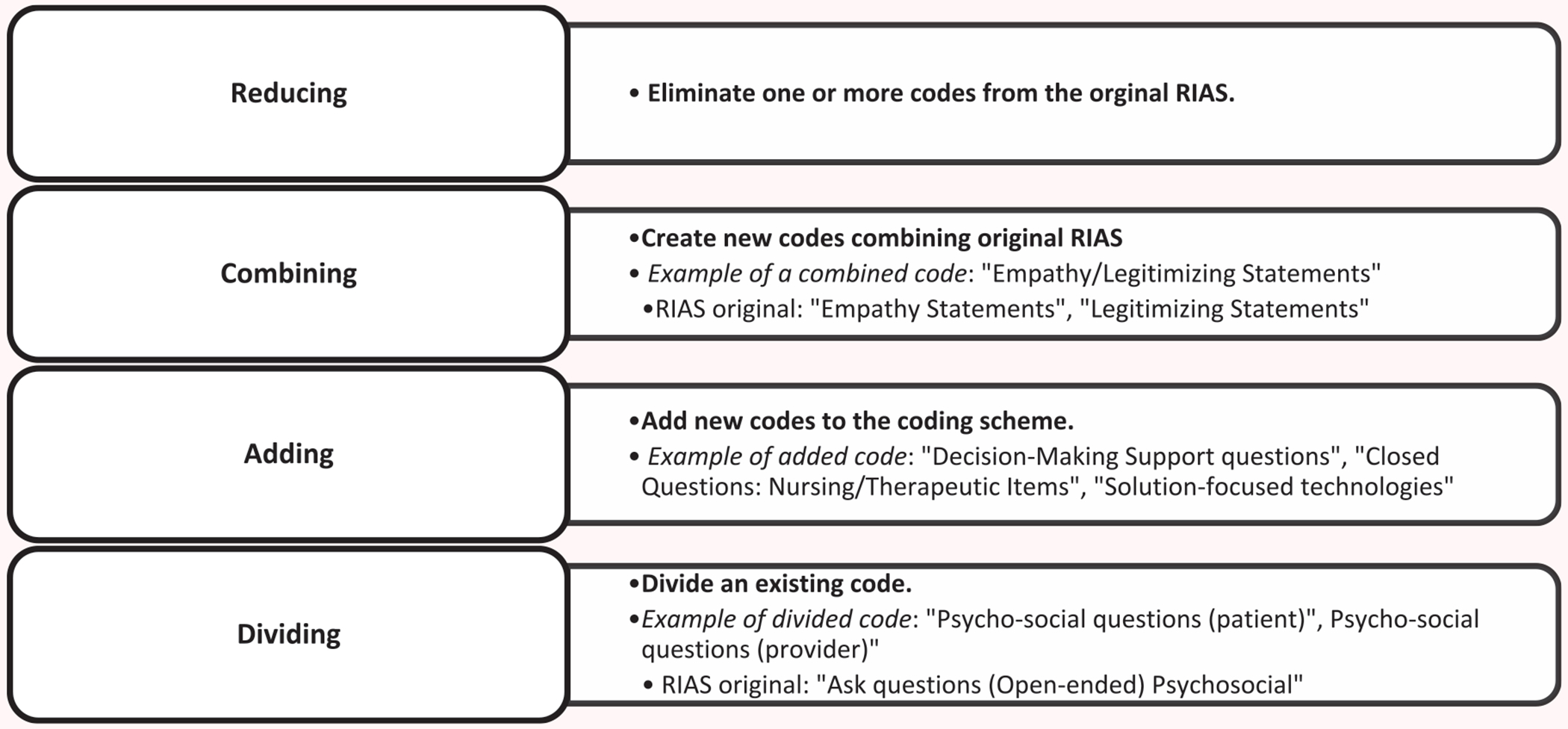
Classification of the main modifications.

Among the 27 articles, 21 reported modifications to the original RIAS. We identified four main types of modifications: a) Reduction: eliminating one or more existing RIAS codes, b) Code aggregation (combining): merging existing RIAS codes, or sometimes together with new codes. Aggregation preserves the original coding concepts but reorganizes them, c) Addition: introducing entirely new codes to capture communication behaviors or interaction types not represented in the original scheme, d) Dividing: splitting an existing code into multiple separate codes to allow for more detailed or nuanced analysis (Fig. 2). Six articles did not mention the type of modification [11,14–16,31,36].

A total of 159 new codes were added in the modified codebooks. The number of codes remaining in the modified RIAS scales was 23 [5–47]. Seventy-one percent of the new codes fall under the task-focused category, and 28% were classified as socioemotional. Of the task-focused codes, the largest proportion (36 out of 114, or 32%) related to questions within the biomedical/medical, general/functional, lifestyle, and other domains. Several new codes were added and slightly modified from the original RIAS categories to enhance specificity. These additions included more detailed closed questions classified under *Nursing/Therapeutic Items* and *Check (assessing knowledge/beliefs)*, reflecting a refined focus on assessing understanding and therapeutic communication (Table 3).

The largest subset of new socioemotional codes (16 of 45; 35.6%) was related to responsiveness, whereas the original RIAS framework included only four responsiveness-related codes. Additionally, the new codes capture aspects of positive and negative affect and emotional expression, which are not addressed in the original framework (Table 4).

Most added codes were introduced to adapt the RIAS to specific study settings, reflecting the need to capture context-specific communication. In contrast, reduced codes were often implemented for practical considerations, such as simplifying the coding process or summarizing dialogue patterns, though some reductions also occurred to reflect study-specific objectives or scope expansions. A detailed list of codes that have been changed is presented in Appendix A.

The codes most frequently retained from the original RIAS were: “Personal Remarks/Social Conversation” was retained in 14 articles, “Shows Agreement or Understanding” (13 articles), and “Shows Concern or Worry” (12 articles). In contrast, the codes most frequently removed or modified, appearing in the fewest articles, were “Asks Questions (Open-ended) – Therapeutic Regimen” (4 articles), “Asks Questions (Closed-ended) – Medical Condition” (4 articles), and “Asks Questions (Closed-ended) – Therapeutic Regimen” (4 articles) (Table 5).

#### 3.2.3. Psychometric evaluation

Evaluation of the modified RIAS codebooks was mainly limited to post-implementation intercoder reliability. Fifteen studies (55.5%) reported varying levels of intercoder reliability using measures such as Cohen’s kappa, Pearson’s correlation coefficients, Krippendorff’s alpha, and percentage agreement. Kappa values ranged from 0.52 to 1.00, Pearson correlation coefficients ranged from 0.69 to 0.98, and reported agreement percentages were generally high (88%–95%). One study [30] used Krippendorff’s alpha, reporting an average of 0.79, and another noted differences in agreement across participant roles (e.g., midwife = 0.67; client = 0.53; partner = 0.82). One article mentioned that intracoder reliability was not measured because the coder was experienced [36]. Only two studies explicitly reported psychometric evaluation before implementation of the modified codebook [24,25] (Table 6).

## 4. Discussion and conclusion

### 4.1. Discussion

This study identified and described published modifications of RIAS, a coding scheme for interpersonal medical communications with widespread utility in diverse settings [4]. Our scoping review found 27 studies that modified the RIAS coding scheme. Overall, the objective was largely achieved: we were able to summarize the types of modifications, the contexts in which they were applied, and the reporting practices of the primary studies.

Our review highlights several key patterns. Almost half of the studies provided no rationale for the modification. The studies did not consistently report a list of codes in the final modified codebook. Many studies justified their modifications, such as reducing, combining, or adding codes, by referencing previous research or citing specific characteristics of the research context, including technology use, therapeutic settings, or veterinary contexts. However, step-by-step descriptions of coder management were often unclear. Modifications to the coding process, including coder training and management, were often insufficiently described. While 15 studies reported calculating inter-rater reliability, we did not find evidence of systematic evaluation or reflection on the modification process itself. These findings suggest that while RIAS is adaptable, inconsistent reporting limits the reliability and comparability of results across studies.

#### 4.1.1. Limitations

While our review provides a novel insight into the modification of a widely used coding framework for doctor-patient communication and the need for more rigor, it is not without limitations.

We did not assess study quality, limiting the interpretation of the quality of each study. However, the lack of detail in many papers would have made it challenging to evaluate quality. We limited our search to databases, bibliographies, and experts, but we may have missed additional studies reported in gray literature. These limitations should be considered when interpreting our conclusions and could be addressed in future systematic reviews.

#### 4.1.2. Prospects for action

Our review highlights the importance of transparency and methodological rigor in reporting measure modifications, coder training, and coding processes. Researchers and journal editors should prioritize clear reporting of modifications to RIAS or other health communication tools, coder training, and coding procedures. We recommend that studies including modified measures document: (a) the original measure’s features requiring modification, (b) the sources informing the modifications, (c) the specific types of modifications made, and (d) how the modified measure was tested for psychometric adequacy, including results. [37] By adopting these practices, future research will improve replicability, strengthen methodological consistency, and provide a more reliable foundation for interpreting interpersonal communication data across diverse clinical contexts.

### 4.2. Conclusion

To our knowledge, no study has systematically evaluated the modifications or quality of changes to RIAS. While the literature includes reviews on modifications to interventions and healthcare guidelines, there is a substantial body of work on the socio-cultural and linguistic modification of measurement scales [38]. Rey Velasco et al. [39] identified and evaluated 11 coding tools (including RIAS) for healthcare interactions, particularly their suitability for written communication. The findings showed that most existing tools rely on categorizing conversations based on interpreted behaviors, themes, or topics, rather than linguistic or socioemotional features. The study also noted inconsistencies in the terminology used for coding categories, concluding that there is a clear need for more studies that apply oral communication coding tools to explore appropriate adaptations. Consistent with these findings, our scoping review revealed significant variability in the codes, with 159 new codes introduced across the 27 articles we analyzed.

Stewart et al. [40] reviewed modifications to health disparity and minority aging measures and found that methodological discussions on modifying measures are rare. Modifications are typically described briefly in the measures section of papers, making them difficult to identify through keyword searches.

Another review [37] assessed the methods used for modification and the quality of health-related guideline modifications. The study identified 72 adapted guidelines, most of which were published by professional societies along with their source guidelines; notably, about one-fifth of the adapted guidelines provided no details about their modification methodology. Our review aligns with previous findings and reinforces the need for transparent reporting. Among those that did, the majority did not follow a published modification method, highlighting a lack of standardization and transparency in the modification process. This limitation raises concerns about the validity and reliability of the modified tools, particularly whether they accurately and consistently measure the intended constructs.

Notably, one study in our review highlighted a key limitation of RIAS. Although it captures both communication content and process, it may fall short in representing the co-construction of meaning during clinical encounters [31]. The frequency of specific communication behaviors does not necessarily equate to meaningful interaction. This observation underscores the importance of considering the limitations of RIAS in fully capturing the collaborative and interpretive nature of healthcare communication.

### 4.3. Practice implications

For future studies employing RIAS, we recommend that researchers provide enhanced clarity regarding any modifications made. Specifically, studies should explicitly articulate the purpose behind any modifications to RIAS, accompanied by a clear list of the utilized codes and categories. Additionally, we suggest providing detailed information about training coders and collaborative processes to maintain coding reliability. Transparency in these methodological practices is crucial for improving reproducibility, strengthening the scientific utility of RIAS-based research, and ensuring that resulting data can be confidently used to guide evidence-informed policy decisions. Acknowledging these limitations and incorporating these recommendations in future research can contribute to the continued refinement and advancement of studies utilizing RIAS.

## Supplementary Material

1

## Figures and Tables

**Table 1:** Summary of the included articles.

#	Author	Title	Year	Country	Setting	Purpose of the article/study
1	Kalet et al. [11]	How well do faculty evaluate the interviewing skills of medical students?	1992	USA	General setting (Medical school evaluation)	To assess reliability and validity of using medical school faculty to assess medical student interviewing skills
2	Hampson et al. [12]	Patient-physician interactions in diabetes management: consistencies and variation in the structure and content of two consultations.	1996	USA	Specialty setting (Diabetes)	Composites were created to allow for coding of patient and physician separately regarding specific tasks as well as coding diabetes-specific content of encounter
3	Caris-Verhallen et al. [13]	Nurse-elderly patient communication in home care and institutional care: an explorative study.	1998	The Netherlands	Specialty setting (Nursing home consultations)	To adapt from analyzing physician-patient communication to nurse-patient communication
4	Caris-Verhallen et al. [14]	Non-verbal behaviour in nurse-elderly patient communication.	1999	The Netherlands	Specialty setting (Nursing home consultations)	To explore the occurrence of non-verbal communication in nurse-elderly patient interaction in two different care settings: home nursing and a elderly patient interaction in two different care settings: home nursing and a home for the elderly
5	Kim et al. [15]	Client communication behaviors with health care providers in Indonesia.	2001	Indonesia	Specialty setting (Family planning)	To analyze active patient communication during family planning consultations
6	Van Weert et al. [16]	Interdisciplinary preoperative patient education in cardiac surgery.	2003	The Netherlands	Specialty setting (Cardiac surgery)	To analyze gaps/inconsistencies/overlaps in preparatory info given by MD, RN and health educators
7	Farquharson et al. [17]	Health beliefs and communication in the travel clinic consultation as predictors of adherence to malaria chemoprophylaxis.	2004	England	Specialty setting (Travel clinic consultation)	To understand adherence to health beliefs, communication during a consultation in a travel clinic and the impact of the consultation in changing health beliefs.
8	Shaw et al. [18]	Use of the Roter interaction analysis system to analyze veterinarian-client-patient communication in companion animal practice	2004	Canada	Specialty setting (Veterinarian-client-patient communication)	To determine the distribution and balance of talk between veterinarian, and client, client and veterinarian, and veterinarian and pet during an appointment
9	Nelson et al. [19]	Adapting the Roter interaction analysis system for telemedicine: lessons from four specialty clinics.	2005	USA	General setting (Telemedicine consultations)	For telemedicine encounters, technology specific subcategories are included.
10	Pieterse et al. [20]	Communication in cancer genetic counselling: does it reflect counselees’ previsit needs and preferences?	2005	The Netherlands	Specialty setting (Oncology)	To describe counselor-counselee interaction during initial cancer genetic counseling consultations and examine if communication reflected counselees’ previsit needs
11	Pieterse et al. [21]	Tailoring communication in cancer genetic counseling through individual video-supported feedback: a controlled pretest-posttest design.	2006	The Netherlands	Specialty setting (Oncology)	To assess the influence of a video-feedback training for cancer genetic counselors on the interaction during initial visits.
12	Katz et al. [22]	Patient literacy and question-asking behavior during the medical encounter: a mixed-methods analysis.	2007	USA	General setting (General practice consultations)	To analyze how literacy affects patient participation in encounter
13	Ellington et al. [23]	Poison control center communication and impact on patient adherence.	2008	USA	Specialty setting (Poison control)	To analyze the effect of communication on adherence to recommendations
14	Mjaaland et al. [24]	Frequency of General Practitioner (GP) communication addressing the patient’s resources and coping strategies in medical interviews: a video-based observational study.	2009	Norway	General setting (General practice consultations)	To analyze the extent of GP discussion of patient coping skills and resources.
15	Gilbert et al. [25]	Communication and outcomes of visits between older patients and nurse practitioners.	2009	USA	General setting (Older adult patients)	To evaluate how content and relationship components of nurse practitioner/older patients communication contribute to patient’s proximal outcomes (satisfaction and intention to adhere) and longer-term outcomes (changes in presenting problems, physical health and mental health).
16	Nelson et al. [26]	Reliability associated with the Roter Interaction Analysis System (RIAS) adapted for the telemedicine context.	2010	USA	General setting (Telemedicine consultations)	To compare tech-related utterances vs non-tech in telemedicine encounters
17	Vegni et al. [27]	A quantitative approach to measure occupational therapist-client interactions: a pilot study.	2010	Italy	Specialty setting (Occupational therapist)	To quantitatively describe the occupational-client interaction by analyzing a role-playing context of occupational therapist and client interactions
18	Shaw et al. [28]	Further validation of the Patient-Practitioner Orientation Scale (PPOS) from recorded visits for back pain.	2012	USA	Specialty setting (Low back pain)	To assess the validity of the PPOS self-report measure by measuring the actual behavior of patients and providers through verbal exchanges using adapted RIAS
19	Kanji et al. [29]	Effect of veterinarian-client-patient interactions on client adherence to dentistry and surgery recommendations in companion-animal practice	2012	Canada	Specialty setting (Dental and surgery)	To analyze veterinarian-client interactions and adherence to dental and surgery recommendations
20	Yoo et al. [30]	Patient-clinician mobile communication: analyzing text messaging between adolescents with asthma and nurse case managers.	2015	USA	General setting (Mobile consultations)	To explore the content of text messaging between clinicians and adolescents with asthma
21	Martin et al. [10]	Antenatal counselling for congenital anomaly tests: an exploratory video-observational study about client-midwife communication.	2015	The Netherlands	Specialty setting (Antenatal care)	To analyze three function antenatal counseling
22	Reblin et al. [31]	Caregiver, patient, and nurse visit communication patterns in cancer home hospice.	2016	USA	Specialty setting (Oncology)	To understand what impacts triadic and dyadic health communication outside of the clinic
23	Driesenaar et al. [32]	Communication during counseling sessions about inhaled corticosteroids at the community pharmacy	2016	The Netherlands	Specialty setting (Chronic obstructive pulmonary disease consultations)	To investigate how pharmacists and pharmacy technicians communicate with patients with asthma and/or chronic obstructive pulmonary disease
24	Menendez et al. [33]	Patients With Limited Health Literacy Ask Fewer Questions During Office Visits With Hand Surgeons.	2016	USA	Specialty setting (Hand surgery)	To analyze the effect of health literacy on patient question-asking
25	Schopf et al. [34]	Physicians’ Reactions to Patients Taking a Position: Sequence Analysis of Admission Interviews in Orthopedic Rehabilitation.	2017	Germany	Specialty setting (Orthopedic rehabilitation and cancer)	To assess the influence of patients expressing opinions on subsequent discussion/shared decision-making
26	Jenson et al. [35]	Patient-centered communication of community treatment assistants in Tanzania predicts coverage of future mass drug administration for trachoma.	2018	Tanzania	Specialty setting (Infection treatments)	To analyze gender differences in patient-centered communication about administering antibiotics for trachoma
27	Pereira et al. [36]	The use of the roter interaction analysis system in assessing veterinary student clinical communication skills during equine wellness examinations in rural Kentucky, USA: A pilot study.	2021	USA	Specialty setting (Veterinarian)	To add to the literature regarding veterinary communication skills and evidence surrounding the vet-client relationship

**Table 2:** Modification to RIAS.

	Author	Modification rationale provided	Purpose of modification	Describes the modification process	Type of modification	Describes the modifications made	Final number of codes listed in the article (total)
1	Kalet et al. [11]	Yes	Scope Expansion (to assess nonverbal behaviors and patient-problem-specific content)	No	Not mentioned	No	21
2	Hampson et al. [12]	No	Not mentioned	No	Combined codes	Yes	7
3	Caris-Verhallen et al. [13]	Yes	Study Setting (to be suitable for analyzing nurse–patient communication)	No	Combined codes	Yes	24
4	Caris-Verhallen et al. [14]	No	Not mentioned	No	Not mentioned	Yes	6
5	Kim et al. [15]	No	Not mentioned	No	Not mentioned	No	25
6	Van Weert et al. [16]	No	Not mentioned	No	Not mentioned	No	32
7	Farquharson et al. [17]	No	Not mentioned	No	Combined codes	Yes	6
8	Shaw et al. [18]	Yes	Study Setting (veterinary setting, conversation in three directions)	No	Added codes/combined codes/reduced codes	Yes	38
9	Nelson et al. [19]	Yes	Study Setting (telemedicine encounters; technology-specific subcategories included)	No	Added	Yes	41
10	Pieterse et al. [20]	No	Not mentioned	No	Combined codes/reduced codes	Yes	17
11	Pieterse et al. [21]	Yes	Scope Expansion (to code the exchange of pedigree data, family medical information, and agenda)	No	Combined codes/reduced codes	Yes	15
12	Katz et al. [22]	Yes	Practical Considerations (to maximize RIAS usefulness for coding patient questions)	No	Added codes / combined codes/reduced codes	Yes	12
13	Ellington et al. [23]	Yes	Practical Considerations (to summarize dialogue patterns)	No	Combined codes	Yes	4
14	Mjaaland et al. [24]	Yes	Scope Expansion (to capture patient resources not emphasized in the original RIAS)	Yes	Combined codes/reduced codes	Yes	14
15	Gilbert et al. [25]	No	Not mentioned	Yes	Combined codes/reduced codes	Yes	33
16	Nelson et al. [26]	Yes	Purpose of the Study (no additional details provided)	No	Added codes	Yes	13
17	Vegni et al. [27]	Yes	Study Setting (role-playing in occupational therapy context)	No	Added codes	Yes	44
18	Shaw et al. [28]	No	Not mentioned	No	Combined codes	Yes	8
19	Kanji et al. [29]	No	Not mentioned	Yes	Added codes	Yes	43
20	Yoo et al. [30]	Yes	Study Setting (to analyze content of text messages between clinicians and adolescent patients using mobile technologies)	No	Added codes /reduced codes	Yes	17
21	Martin et al. [10]	No	Not mentioned	No	Added codes	Yes	16
22	Reblin et al. [31]	Yes	Scope Expansion (to capture co-creation of meaning in communication)	No	Not mentioned	No	8
23	Driesenaar et al. [32]	Yes	Purpose of the Study (no additional details provided)	No	Added codes/divided codes	Yes	17
24	Menendez et al. [33]	Yes	Practical Considerations (to maximize usefulness and practicality)	No	Reduced codes	Yes	11
25	Schopf et al. [34]	No	Not mentioned	No	Added codes/divided codes	Yes	44
26	Jenson et al. [35]	Yes	Practical Considerations (to simplify and better capture the study’s purpose)	No	Combined codes/reduced codes	Yes	8
27	Pereira et al. [36]	Yes	Study Setting (veterinary context)	No	Not mentioned	Yes	13

**Table 3:** New codes added to the task-focused Category.

Main category	Themes	Codes
Questions	**Biomedical/** **Medical**	Closed Questions: Nursing/Therapeutic Items; Open Questions: Nursing/therapeutic items; Health education questions; Decision-Making Support questions; Biomedical questions; Questions about medication; Medical questions about surgery; Other medical questions; Medical questions; Biomedical open-ended questions; Biomedical close-ended questions; Nonmedical questions; Biomedical information Closed-ended questions; Biomedical information Open-ended questions
	**General/** **Functional**	Question asking; Asks Questions (Closed)-TECH; Asks Questions (Open)-TECH; Direct requests for questions; Open-ended questions; Close ended questions; Asks Questions; Questions about ward; Questions about preparation; Questions about stay after surgery; Practical questions about surgery
	**Lifestyle**	Questions about lifestyle and psychosocial issues; Questions about lifestyle (patient); Psycho-social questions (patient); Psycho-social questions (provider); Lifestyle Questions (provider); Lifestyle and social information Closed-ended questions; Lifestyle and social information Open-ended questions
	**Other**	Asking open-ended technological questions; Asking open-ended intervention-related questions; Asking closed-ended technological questions; Asking closed-ended intervention-related questions
Information	**Biomedical/** **Medical**	Information about nursing/therapeutic items; Decision-Making Support information; Nonmedical information; Medical information; Biomedical information bid for repetition; Biomedical information giving; Biomedical information; Information about medication; Biomedical information (patient); Medical information about surgery; Other medical information
	**General/** **Functional**	Practical information about surgery; Practical information from relatives; Information about today’s program
	**Lifestyle**	Information about lifestyle and psychosocial issues; Psycho-social information (patient); Gives Occupational Therapy Information; Asks Occupational Therapy Information; Gives Occupational Therapy Information Lifestyle; Asks Occupational Therapy Information Lifestyle; Giving psychosocial information; Lifestyle and social information giving
	**Other**	Gives information- TECH; Information provision; Gives information; Giving technological information; Giving intervention-related information; Lifestyle; General information about ward.
Education	**Education/** **Counseling**	Health education information; Decision Making support counseling; Family education; Medical education; Psychosocial education; Counsels or directs behavior- TECH; Biomedical information counseling; Lifestyle and social information counseling; Counsels direct behavior; Psychosocial info and counseling; Biomedical Education and counselling; Lifestyle Education and counselling
	**Orientation**	Gives direction; Gives orientation; Gives orientation, instructions- TECH
Comprehension checking	**Checking/** **Facilitative**	Resources; Offer the possibility to talk about antenatal tests again; Attribution; Facilitation and patient activation; Check (assessing knowledge/beliefs); Paraphrase/checks understanding-TECH; Bids for Repetition-TECH; Procedural Talk; Clarification; Opinion (ask/give); Provides instruction; Paraphrase/Checks for understanding medical; Paraphrase/Checks for understanding psychosocial; Seeks understanding.
Other		Medical; Therapeutic; Lifestyle/social; Other; Coping; Solution-focused technologies; Medical history taking; Preparation for surgery; Hospital stay after surgery; Lifestyle advice; Procedural Language; Nonmedical/procedural; Medical composite; Diet Talk; Discussion related to Dental Care (proficiency code); Discussion related to Surgical Care (proficiency code); Other (provider); Other (patient); Other; Residual, unintelligible

**Table 4:** New codes added to the socioemotional category.

Themes	Codes
Emotional expression Relational talk and opinions	Emotional talk; Psychosocial/Feelings; Emotional support Patient Expression of Opinion; Verbal attention; Social talk; Social talk (provider); Social talk (patient); Activating and partnering; Partnership building- TECH; Dominance/assertiveness; Seeks opinion
Responsiveness	Accordance; Facilitation; Empathy; Cues of Interest; Empathy -TECH; Shows concern or worry-TECH; Reassures, encourages, shows optimism- TECH; Legitimizes- TECH; Asks for reassurance- TECH; Asks/gives reassurance; Rapport building; Positive Talk; Interest/concern; Friendliness/warmth; Encouragement; Reassurance
Positive affect	Affirmative head nodding; Smiling; Eye contact; Body position; Gestures; Affective touch; Instrumental touch
Negative affect	Contradiction; Shows disapproval; Negative Talk; Anger/irritation; Anxiety/nervousness; Concern; Concern, reassurance; Disagreement
Other	Data gathering; Forward leaning

**Table 5:** Frequency of Original RIAS Codes Across Articles.

	Codes	Number of articles using the code
Most codes used	Personal Remarks, Social Conversation	14
	Shows Agreement or Understanding	13
	Shows Concern or Worry	12
	Asks for Reassurance	12
	Paraphrase/Checks for understanding, accuracy, confirmation, clarification	12
	Asks for Understanding	12
	Gives Orientation, Instructions	11
	Shows Approval-Direct	10
	Shows Disagreement/Disapproval- Direct	10
	Partnership Statements (Provider category)	10
Fewer codes used	Asks Questions (Closed-ended)- Psychosocial	6
	Counsels- Medical Condition/Therapeutic regimen (Physician only)	6
	Asks Questions (Closed-ended)- Medical condition	5
	Asks Questions (Closed-ended)- Other	5
	Asks Questions (Open-ended)-Other	5
	Gives information- Therapeutic regimen	5
	Asking for Permission (Physician Category)	4
	Asks Questions (Closed-ended)- Therapeutic regimen	4
	Asks Questions (Open-ended)- Medical condition	4
	Asks Questions (Open-ended)-Therapeutic regimen	4

**Table 6:** Psychometric Evaluation.

	Author	Psychometric Evaluation Before Use	Post-Implementation Psychometric Evaluation descripted
1	Kalet et al. [11]	Not mentioned	Intercoder reliability, Cohen’s Kappa: 1.0
2	Hampson et al. [12]	Not mentioned	Pearson correlation coefficient between the two coders: Asks Questions, r = 0.95; Gives Information, r = 0.97, Positive Talk, r = 0.97, Social Conversation, r = 0.83, Partnership Building, r = 0.72; and Diet Talk, r = 0.97. For the patient composite variables, inter-coder reliabilities were as follows: Asks Questions, r = 0.92, Gives Information r = 0.93, Positive Talk, r = 0.97; Social Conversation, r = 0.93, and Diet Talk, r = 0.94
3	Caris-Verhallen et al. [13]	Not mentioned	Intercoder reliability, Pearson’s r between 0.69 and 1.00
4	Caris-Verhallen et al. [14]	Not mentioned	Intercoder reliability, Pearson’s R proved to be between 0.70 and 0.98
5	Kim et al. [15]	Not mentioned	Not mentioned
6	Van Weert et al. [16]	Not mentioned	Intercoder reliability, Cohen’s Kappa: 0.78
7	Farquharson et al. [17]	Not mentioned	Not mentioned
8	Shaw et al. [18]	Not mentioned	Not mentioned
9	Nelson et al. [19]	Not mentioned	Not mentioned
10	Pieterse et al. [20]	Not mentioned	Intercoder reliability, Cohen’s Kappa: 0.69
11	Pieterse et al. [21]	Not mentioned	Intercoder reliability, Cohen’s Kappa: 0.69
12	Katz et al. [22]	Not mentioned	Not mentioned
13	Ellington et al. [23]	Not mentioned	Intercoder reliability, Pearson correlation coefficients averaged ≥ 0.70 Intercoder reliability, 90% congruence regarding total number of utterances and each of the categories
14	Mjaaland et al. [24]	Yes, Kappa.	Intercoder reliability, Cohen’s Kappa: 0.52
15	Gilbert et al. [25]	Yes, Pearson correlation coefficient: r = .85	Not mentioned
16	Nelson et al. [26]	Not mentioned	Intra-class correlation coefficient for each category and for the following summary clusters: socioemotional exchange, task-focused exchange, technology-related exchange, and grand total (values less then 0.4 are thought to reflect poor reproducibility, values between 0.4 and 0.75 fair to good reproducibility, and values exceeding 0.75 excellent reproducibility
17	Vegni et al. [27]	Not mentioned	Not mentioned
18	Shaw et al. [28]	Not mentioned	Intercoder reliability, 88% for provider utterances and 91% for patient utterances provider utterances and 91% for patient utterances
19	Kanji et al. [29]	Not mentioned	Not mentioned
20	Yoo et al. [30]	Not mentioned	Intercoder reliability corrected for agreement by chance (Krippendorff’s alpha) indicated an average reliability of 0.79 across all coding categories
21	Martin et al. [10]	Not mentioned	Intercoder reliability for the midwife (0.67), the client (0.53), and partner categories (0.82)
22	Reblin et al. [31]	Not mentioned	Intercoder reliability, Cohen’s Kappa: 0.65
23	Driesenaar et al. [32]	Not mentioned	Intraclass correlation coefficient reliability averaged 0.85
24	Menendez et al. [33]	Not mentioned	Intercoder reliability, 90% agreement
25	Schopf et al. [34]	Not mentioned	Intercoder reliability, Cohen’s Kappa: 0.68
26	Jenson et al. [35]	Not mentioned	Intercoder reliability, 91% reliability; 95% agreement
27	Pereira et al. [36]	Not mentioned	Intracoder reliability was not measured because coder was experienced

## Appendix A. Supporting information

Supplementary data associated with this article can be found in the online version at doi:10.1016/j.pec.2026.109566.
